# The move to open: medical library leadership in scholarly communication*

**DOI:** 10.5195/jmla.2021.1127

**Published:** 2021-01-01

**Authors:** Chris Shaffer

**Affiliations:** 1 chris.shaffer@ucsf.edu, University Librarian, Assistant Vice Chancellor for Academic Information Management, and Adjunct Professor, Department of Medicine, School of Medicine, University of California, San Francisco, CA

## Abstract

Over the years, health sciences librarians have been change agents, leading the charge on issues of importance to the profession and the communities we serve. From its founding in 1898 with the Exchange, the Medical Library Association (MLA) has been dedicated to improving access to health information. In 2003, the Board of Directors published a statement supporting open access to information generated from federally funded scientific and medical research and maintained that having access to timely, relevant, and accurate information is vital to the health of the nation and its education and research programs. At some financial risk, the association made the *Journal of the Medical Library Association (JMLA)* open access and published the entire archive of *JMLA* and its predecessor, the *Bulletin of the Medical Library Association,* in PubMed Central. Nearly two decades later, the promise of open access and open science finally seems to be coming to fruition. In the 2020 Janet Doe Lecture, Chris Shaffer, AHIP, described the ways that MLA has led the profession, standing behind a shared vision and “walking the walk.” In challenging listeners to embrace open science, he affirmed that, as leaders in improving access to health sciences information since 1898, medical librarians must work in the open science arena to realize our vision “that quality information is essential for improved health.”

**Figure:**
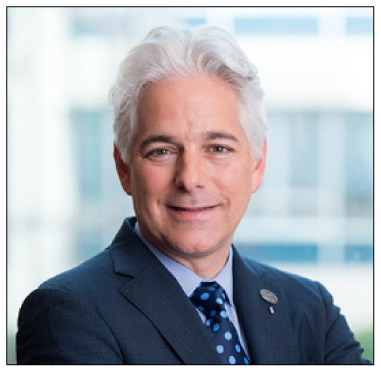
Chris Shaffer

## INTRODUCTION

Thank you for the gracious introduction, Gerald (Jerry) Perry, AHIP, FMLA. As a queer librarian living in San Francisco, it was very gratifying to learn more about the work that you did to support the communities and individuals who suffered during the HIV/AIDS pandemic in your lecture, “The Activist Health Sciences Librarian” [1]. As a child of the 1960s and 1970s who grew up listening to the protest songs of folk music, I was inspired by my mentor Elaine Russo Martin, FMLA, who called us to embrace social justice medical librarianship in her Janet Doe Lecture at the 2018 annual meeting [2]. And as someone whose motto in life is “subvert the dominant paradigm,” I was moved by Ana D. Cleveland, AHIP, FMLA, when she asked us, in her 2010 Janet Doe Lecture, to reimagine the pathways of health sciences library education in response to changing paradigms and trends in health care and health information [3].

Each of you has influenced my work as a librarian from the very beginning of my career. Dr. Ana introduced me to medical librarianship in graduate school at the University of North Texas; Elaine was director of the Library of the Health Sciences at the University of Illinois at Chicago, where I took my first job following library school; and my “frolleague” Jerry welcomed me to the LGBT Special Interest Group (the “SIG”) at my first Medical Library Association (MLA) meeting. I am honored to follow in your footsteps and those of the many wonderful librarians who have stood before me at previous annual meetings to speak on the history or philosophy of medical librarianship.

Like so many other Janet Doe Lecturers, I am neither a philosopher nor a historian. While I have an undergraduate degree in philosophy with a minor in history from Texas A&M University, I can only claim to be a dabbler in the study of the past and of the fundamental nature of knowledge, reality, and existence. Thirty years later, I can hardly remember what I am sure I believed at the time were profound discussions of Willard Van Orman Quine's theories of language and logic or the names of the many usurpers and “False Dmitrys” who claimed the title of tsar in the Russian Time of Troubles. That said, I would be happy to discuss Henry Littlefield's debunked theory that the *Wizard of Oz* is an allegory for monetary policy in nineteenth century America over coffee or cocktails, if you are so inclined.

As I was musing over the issue of selecting a topic for the lecture, I did what so many of my predecessors have done: I read the entire corpus of previous Janet Doe Lectures, and, this being the modern age, I watched a few of the more recent ones on video. I highly recommend this activity for those who want to learn more about our association and profession.

My first meeting was in Kansas City, Missouri, in 1996, where Robert M. Braude, AHIP, FMLA, of the Samuel J. Wood Library at Cornell University Medical College, chose the title, “On the Origin of a Species: Evolution of Health Sciences Librarianship.” He challenged listeners to consider the long history of health sciences communication with these words:

Our territory has been the world of biomedical information, represented for more than 500 years as print on paper. But the reality is that biomedical information has existed for longer than 500 years and in a variety of different containers. The newer containers resulting from developments in information technology just represent a more rapid change in the form and format of this information. Nevertheless, these developments are fundamentally changing the nature of information and its management, which, traditionally, has been our responsibility. [4]

For myself, considering the entire history of biomedical information seemed rather a tall order, so I limited my search to the time since the founding of the Association of Medical Librarians in 1898, which later became MLA. That led me to read Scott Adams's book, *Medical Bibliography in an Age of Discontinuity* [5]. While I recommend it to those who want to explore our history, it is, shall we say, quite dry. Finally, Jennifer Connor's excellent book, *Guardians of Medical Knowledge: The Genesis of the Medical Library Association* [6], written in celebration of the 100th anniversary of MLA, was recommended to me by Mary Langman from MLA headquarters. In it, Connor quotes Dr. George M. Gould's address at the first MLA meeting:

I look forward to such an organization of the literary records of medicine that a puzzled worker in any part of the civilized world shall in an hour be able to gain a knowledge pertaining to a subject of the experience of every other…in the world. [7]

This quite naturally led me to muse upon my own history and career, which coincided with the development of the Internet and the World Wide Web. Sadly, Gould's vision has not yet been realized, despite the promise of technology.

I am sure that I am similar to many of you in reminiscing over fond memories of our school and public libraries and the librarians who served and influenced us. However, it is the rare librarian I have met who can claim to be “second generation.” As the son of a serials librarian, I spent my childhood ensconced in academic libraries, where I whiled away my time reading decades of *Mary Worth, Alley Oop,* and other serial comics on microfilm. I learned about multiple types of libraries, as the university where I grew up had its roots as a teacher's college and contained a standalone curriculum library within the main library. In addition, with the advent of automation, the university and local public library created a union catalog using the Northwestern Online Total Integrated System, which some of you may remember as NOTIS, giving me my first experience with library cooperation and automation.

After my undergraduate days at Texas A&M (where I was a frequent visitor to the Cushing Memorial Library and Archives and voracious devourer of titles in the Science Fiction & Fantasy Research Collection, to the detriment of my studies), I became an interlibrary loan (ILL) assistant in the Ralph W. Steen Library at Stephen F. Austin State University in Nacogdoches, Texas, my home town. This was, of course, the same library where my mother had previously worked and in which I spent much of my youth. On the job, I learned my first lessons about eliminating barriers to information access. At the reference desk, I helped undergraduate students navigate *Chemical Abstracts* and other print indexes. I manually entered hundreds, perhaps thousands, of serials holdings into InfoTrac, a CD-ROM article indexing utility, to speed time-to-shelf by allowing filtering on local collections, which were, of course, print in those days. Another project I worked on was an analysis of the cost of administering patron ILL fees. It turned out that it cost us more to process an invoice than the $2 per request we were taking in. Rather than raise the price, the librarians with whom I worked decided to eliminate the charge. The faculty and students were ecstatic.

In the years since, I have worked as an ILL librarian, a reference and education librarian, a technology coordinator for the National Network of Libraries of Medicine, an assistant director for public services, and a university librarian at four academic medical centers. A consistent theme in my career has been providing access to information while living through years of increasing prices and cuts to collections. I cannot count the number of presentations I have given to faculty and students explaining that a subscription to a single year of a journal can cost more than a luxury automobile.

I was already inclined by my upbringing to support the rallying cry “information wants to be free!” Today, I ask you and your fellow medical librarians to commit to open science, defined by the European Union project FOSTER as:

the practice of science in such a way that others can collaborate and contribute, where research data, lab notes and other research processes are freely available, under terms that enable reuse, redistribution and reproduction of the research and its underlying data and methods. [8]

So, back to what I learned from the Janet Doe Lectures, *Medical Bibliography in an Age of Discontinuity, The Guardians of Medical Knowledge*, and a perusal of MLA annual meeting proceedings, Board of Directors reports, and policies.

## THE EXCHANGE

From the beginning, the association has been focused on improving access to health information. Even today, the MLA Vision statement begins with the sentence “The Medical Library Association (MLA) believes that quality information is essential for improved health” [9]. When Gould proposed the formation of “an organization of Medical Librarians” in 1898, he stated:

It seems to me strange beyond all belief in the stage of civilization which we have reached…when every place of human activity has recognized that the *sine qua non* of progress is organization and intercommunication, that the pricelessly precious results of medical knowledge should be given over to the rapine of commercialism, and to the barbarism of unorganization in which our medical libraries at present do not flourish. [7]

He went on to describe eight committees, the first of which would be “On Exchange of Library Duplicates.” In 1901, he described the Exchange of the Association of Medical Librarians as “a sort of literary clearing-house for all its associate members, whereby may be utilized perhaps a million volumes of books and periodicals at present wasted because no mechanism has heretofore existed to act as the intermediary of the distribution” [10].

For those of you who might not be familiar with it, the Exchange was one of the things that set MLA apart from similar organizations, such as the American Library Association (ALA), founded in 1876. Management of the Exchange was a constant topic of business at annual meetings for decades, and there were many articles about it in the *Bulletin of the Medical Library Association (BMLA),* now the *Journal of the Medical Library Association (JMLA).* As recently as the 1982 annual meeting, the work of the Exchange was such that the Exchange Committee held two meetings (one at the dreaded hour of 7:00 a.m.!) [11].

When I was involved in revising the Bylaws in 2016, I learned that the Exchange was required in the Bylaws and specified in the Certificate of Incorporation [12]. As the first major initiative of our association to improve access to health information, the Exchange continues today, having merged with the former Swets Blackwell BackMed email list in 2015 [13]. I would be remiss, however, to overlook the fact that Connor described membership in the association, and thus access to the Exchange, as a tool of oppression. As she noted, “personal, public, sectarian, commercial, allied science, and the then-termed ‘colored' medical school libraries all were denied membership” [14].

## COST OF JOURNALS

Another challenge facing medical librarians, from the very beginning of the association, was a shortfall in funding contrasted with the ever-increasing cost of resources. Of the eight committees proposed by Gould in his 1898 invitation, four were concerned with acquiring materials by donation or at low cost, and another “For Securing Endowments of Medical Libraries” [7]. To support the proposed committee “For Securing and Distribution of Transactions of Medical Societies,” you might be interested to learn that, beginning in MLA's founding year of 1898, each member received a free subscription to the *Journal of the American Medical Association.* Unfortunately, this lovely tradition of free journals ended in 1904 and has not yet resumed [6].

From early in the association's history, medical librarians have served important roles in addressing issues of cost. In her 1982 Janet Doe Lecture, Ursula H. Poland, FMLA, described efforts to make abstracts affordable and improve their quality. She recounted that Eileen R. Cunningham, FMLA, and Janet Doe, FMLA, as delegates from MLA to the first national conference of the US National Commission on the United Nations Educational, Scientific and Cultural Organization (UNESCO) in 1947, were instrumental in a resolution that led UNESCO and the World Health Organization (WHO) to establish a Coordinating Committee on Abstracting and Indexing of Medical and Biological Sciences. One of the key issues was the high cost of abstracting journals, which led Cunningham to advocate for authors' abstracts and the reduction of duplication in abstracting. They were also very involved in the subsequent 1948 UNESCO Conference of the coordinating committee, which led to a resolution that “*Excerpta Medica* incorporate itself into a not-for-profit foundation instead of a commercial enterprise” [15]. I expect many of you, like me, would be surprised to learn that the precursor organization that led to EMBASE was a nonprofit foundation! Gertrude L. Annan, FMLA, in the first Janet Doe Lecture, said: “In 1967 as we meekly accept spiraling prices, often with a burdensome higher pricing for libraries, Mrs. Cunningham's persistence seems tilting at windmills” [16].

These concerns continue to the present day, of course. In 1987, the MLA Board of Directors issued a resolution regarding the cost of foreign periodicals “that the Medical Library Association deplores and protests said pricing differential and urges those publishers involved to immediately eliminate market based discriminatory pricing” [2020 MLA policies, provided by MLA staff].

## INTERLIBRARY LOAN

I now turn to ILL. It might seem obvious now, but a 1962 study by the National Library of Medicine (NLM) showed that most ILL requests were for common titles, indicating that many health care practitioners and the libraries that served them did not have access to the core literature of health sciences [17]. The history of ILL, particularly for journal articles, has been full of discussions of copyright and cost.

In 1939, before the advent of the first commercial Xerox machine (which came in 1959), medical librarians began grappling with the challenge of microfilm, as reported by Mary A. Bennett and D. H. Litchfield in the *BMLA.* They imagined a world in which a “microphotographer” in New York could take a picture of a book in Spain by means of a “supertelescopic” lens [18]. Later, in 1944, Thomas Keys wrote an editorial urging medical libraries to follow the lead of the Army Medical Library (which became NLM) in providing free microfilm copying in service of “the advancement of research and learning” [19]. This was, of course, an early example of non-returnable copying—for free!

In 1966, just before the establishment of the first Regional Medical Libraries, the Duke University, Wake Forest University, and University of North Carolina (UNC) Chapel Hill medical libraries began using the Teletypewriter Exchange Service (TWX) to transmit ILL requests. For those who are not familiar with it, TWX basically connected a typewriter to a telephone line—more of an advance of the telegraph than a precursor to telefacsimile, or fax machines. This had the advantage of allowing ILL staff to transmit requests without requiring what many reportedly considered the cumbersome ALA ILL form.

It also significantly decreased the turnaround time for ILL. Warren Bird from Duke reported that “the old expected delays of several weeks have been cut to a few days at the most—and a few hours at best—since negative reports are transmitted within twenty-four hours.” Reported advantages included the asynchronous nature of TWX versus telephone and “eliminating misunderstanding when dealing with foreign languages, exotic medical terms, and complex citations.” NLM soon joined the network, which grew to 72 medical libraries by 1968. Interestingly, improving the efficiency of cooperative ILL significantly reduced the number of requests sent to NLM. For the 3 North Carolina libraries, the number of requests filled by NLM dropped from 58% to 32% in just 3 years [20].

With the Medical Library Assistance Act of 1965 began a well-documented era of expansion of new medical libraries in health care settings and a strengthening of academic medical libraries. In 1967, the Regional Medical Library Program, now the National Network of Libraries of Medicine (NNLM), was established. A primary activity of the new network was facilitating ILL in a hierarchical system based on geography. Over 3,000 members were served by more than 100 resource libraries and 11 Regional Medical Libraries (RMLs).

Showing continued interest in copyright as it related to ILL, in 1978, the MLA Board of Directors urged NLM to assist librarians by updating Medical Subject Headings (MeSH) to indicate “a journal's willingness to permit unrestricted photocopying for scientific, educational and research purposes of the material in their publications” [2020 MLA policies, provided by MLA staff]. In 1982, Washington University in St. Louis launched OCTANET, so named because they were the RML for Region VIII. Based on the Periodicals Holdings in Libraries of Schools of Medicine (PHILSOM), developed under the leadership of Estelle Brodman, FMLA, it was perhaps the first online ILL system that used automated routing, an innovation that significantly speeded delivery of materials to patrons [21]. This was the foundation of the DOCLINE system we all use today. When DOCLINE debuted in 1985, six years after OCLC began offering resource sharing, it was notable that it was more than a decade before Colorado State University developed RapidILL in 1997 to enable the same functionality [22]. Henry Lemkau Jr. FMLA, wrote in his 2007 Janet Doe Lecture:

Estelle Brodman's impact on medical librarianship as a scholar, teacher, and researcher were major. Estelle's dissertation, “The Development of Medical Bibliography,” published in 1954 remains a landmark work. She developed the PHILSOM automation project for serials control in the 1960s. This was one of the first applications of the new computer technologies to library routines. [23]

## AUTOMATION

Switching gears from ILL to library automation: I remember getting enormous spools of PHILSOM computer tape from Washington University in St. Louis at the NNLM Greater Midwest Region office in the late 1990s. We converted the holdings to regional and state union lists on microfilm and sent them off to NLM to update SERHOLD.

In the 1950s and 1960s, medical librarians initiated some of the first attempts to automate library indexing and catalogs. A librarian who worked on the Welch Medical Indexing Project for the Welch Medical Library at Johns Hopkins University, Eugene Garfield, FMLA, went on to establish *Science Citation Index,* now part of Web of Science [24]. In his 1984 Janet Doe Lecture, Irwin Pizer, AHIP, FMLA, amusingly recounted that the Columbia-Harvard-Yale Medical Libraries Computerization Project went by the names CoHaYaMed, HYCCUP, and YCH (pronounced yechhh). However, your assumptions about what it was intended to do are likely incorrect, as it actually automated the printing of catalog cards. Unfortunately, while that project was not in the end successful, Pizer went on to say:

there are probably few readers who recall that the genesis of the Ohio College Library Center, now OCLC, programs, and their profound impact on all types of libraries, can be traced directly to the work done by Kilgore *[sic]*, Fleming, and Esterquest in the early 1960s. [25]

Pizer, who was director of the Health Sciences Library at Upstate Medical Center in Syracuse, led the SUNY Biomedical Communication Network as it developed a dictionary of MeSH terms “matched to vernacular terminology” in one of the first online information retrieval services for biomedical literature [25]. The program lasted from 1968–1977 and eventually led to the foundation of Bibliographic Retrieval Services (BRS), which became part of Ovid Technologies.

Around the same time, NLM debuted the Medical Literature Analysis and Retrieval System (MEDLARS) to automate the production of the *Index Medicus,* which also enabled a system named AIM/TWX. Wilhelm Moll, director of the University of Virginia Medical Library, wrote in 1971 that an AIM/TWX trial was quite popular. The system allowed users to search the *Abridged Index Medicus (AIM),* which indexed 100 core clinical titles, via the previously mentioned TWX. Because users found it so difficult to build searches with MeSH, the search strategies were generally designed one day before the search was scheduled to run. Searches were primarily conducted for clinicians, rather than researchers, and as Moll reported, “the overwhelming majority of the searching, however, involved multi-concept requests which was greatly speeded up by the use of the machine” [26]. The success of the trial informed the development of MEDLINE, or “MEDLARS Online.”

In 1986, NLM launched Grateful Med, the first user-friendly front end to ELHILL, the command language for MEDLINE searches. I remember stories of librarians traveling to NLM for five-day ELHILL training sessions. While services such as EasyNet had pioneered end-user access to periodical databases on Dialog, prices were often exorbitant [27]. Grateful Med, though not free, was quite affordable as NLM charged only the actual cost to run the system, and it significantly expanded access to MEDLINE [28]. However, I should note that not everyone agreed that end-user searching was an appropriate step, as evidenced by a letter to the editor in the January 1994 issue of the *BMLA,* which quoted Herbert S. White's previous opinion piece: “Using GRATEFUL MED on your own, in the hope of performing an adequate and cost-effective search, is not only a dumb idea, it is a dangerous idea!” [29, 30]. Ten years later, in 1996, both PubMed and Internet Grateful Med were launched, and end-user searching was here to stay. One year after that, Vice President Al Gore demonstrated them at a press conference to announce free access to MEDLINE via both services.

Back when Yahoo was the preeminent Internet search engine, a group of medical librarians in the Midwest launched HealthWeb in 1994. Representing the Committee on Institutional Cooperation, now known as the Big Ten Academic Alliance, the twelve health sciences libraries created an index of evaluated, annotated Internet resources. Though there was much discussion of assigning MeSH terms or NLM Classification to websites, the project was overtaken by developments in Internet search, notably the rise of Google, before those features could be implemented [31].

## CONSUMER HEALTH INFORMATION

Moving on to consumer health information: as reported by Alan M. Rees in the 1992 Janet Doe Lecture, medical library pioneers in the late 1970s identified the “need to disseminate medical information to the community at large.” He expressed the firm belief that “access to the literature of medicine should not remain the exclusive privilege of health care professionals.” At the time, this was a radical idea that had not yet been fully accepted by medical libraries, many of which he reported “still have a policy of restricted access” [32].

Ruth Holst, AHIP, FMLA, in the 1990 Janet Doe Lecture, informed attendees that “the history of hospital librarianship tells us that service to patients has been with us since the late nineteenth century” and distinguished between the “hospital library,” which was synonymous with “patient library,” and the “medical staff library.” She acknowledged that in the early years much of the service for patients was related to books as a therapeutic tool. However, that changed in the 1970s and 1980s, driven in part by medical librarians in the Veterans Administration [33]. There was much debate “about the librarian's liability in providing information services to the lay public.” However, intrepid medical librarians forged ahead, and in 1981, the Consumer and Patient Health Information Section (CAPHIS) was formed as a provisional section, followed by formal recognition in 1986.

It took twelve years before MLA, in 1998, expanded its book publishing program to include health care professionals and consumers as target audiences. Prior to that, the books program was focused entirely on information professionals. And in 2005, the MLA Board of Directors endorsed the recommendations of the Health Information Literacy Task Force and acknowledged the leading role of CAPHIS in the association's initiatives for patients [34].

All that said, patients and their caregivers still do not have adequate access to the results of health sciences research, and efforts by publishers have come up short. PatientINFORM, from the International Association of Scientific, Technical and Medical Publishers (STM), was an attempt to bring articles to patients and their caregivers that launched in 2005 and folded in 2017. PatientACCESS was a similar initiative from the Copyright Clearance Center. As David Crotty reported in the *Scholarly Kitchen,* both initiatives suffered from a lack of a discovery mechanism [35].

However, publishers have done a better job on the international front. The Hinari Access to Research in Health Programme was established by the WHO in 2001. Part of the Research4Life initiative, Hinari was cofounded by major biomedical publishers in collaboration with the Yale University Library. In 2005, MLA established the Librarians without Borders® E-Library Training Initiative, which “improves access to critically important health information in underserved countries through training in information retrieval and library information assistance.” (Note that a good name never goes to waste, and MLA's program is distinct from Libraries without Borders and the Canadian library student organization also named Librarians without Borders.)

In 2015, MLA awarded the Louise Darling Medal for Distinguished Achievement in Collection Development in the Health Sciences to Hinari, which provides free or low-cost online access to journals to residents of developing countries and whose collection development efforts have been supported by the Harvey Cushing/John Hay Whitney Medical Library at Yale University [36]. However, participation in Hinari is not seamless and must be mediated through registered institutions, which, of course, leaves many health professionals, patients, and their caregivers without access. And while more than 100 countries are eligible, many that you might expect to be eligible are not.

## OPEN ACCESS

And with that, I turn to consideration of MLA's role in promoting open access. You might be wondering why I spent so much time talking about the Exchange, the cost of resources, ILL, automation, and consumer health information. While some of these are more obviously related to open access than others, I hold that the philosophy and values of medical librarianship led our predecessors to do great things in service of access to information. Some of their experiments were more successful than others, but they all represented leadership in the profession toward improving access.

I take this opportunity to remind you of MLA's shared vision “that quality information is essential for improved health,” and add two selections from MLA's statement of core values, which are, and I quote, “our principles, beliefs, ideals, and standards we will not compromise. The heart and soul of our association that provide meaning to our success.” We value:

- Use of scientific evidence in making health care decisions, and
- Public awareness of, access to, and use of high-quality health information. [9]

In 1999, MLA made financial contributions to the Scholarly Publishing and Academic Resources Coalition (SPARC). MLA also contributed to Shared Legal Capability (SLC), now known as the Library Copyright Alliance, whose goal was to exert joint library community positions on key intellectual property issues [2020 MLA policies, provided by MLA staff].

In his inaugural address at the 2000 annual meeting in Vancouver, British Columbia, Canada, J. Michael Homan, AHIP, FMLA, described PubMed Central and PubMed Express and announced that “the *BMLA* will be available in full text online and continue a 100-year commitment to preserving and promoting the best evidence of health sciences librarianship” [37]. Despite the risk to the association of opening its flagship research journal at this early date in the open access movement, MLA “walked the walk.” While it took a little bit longer than Homan predicted, the *BMLA* backfile was published on PubMed Central by 2003. Unfortunately, in the face of strong criticism, in 2001, the National Institutes of Health (NIH) dropped plans for the PubMed Express preprints repository [38].

In the summer of 2002, activist members of MLA challenged the association to reconsider the *JMLA* copyright policy. In response, T. Scott Plutchak, AHIP, FMLA, *JMLA* editor-in-chief at the time, announced a revised copyright policy for *JMLA* to end the practice of requiring that authors transfer copyright, effective with the July 2003 issue [39].

And, in a sign that MLA was starting to seriously pay attention to scholarly communication issues, in 2003, the MLA Board of Directors:

- issued the “MLA Statement on Open Access” [40];
- established the Scholarly Publishing Task Force, which was charged to assess trends and work with the Association of Academic Health Sciences Libraries (AAHSL);
- helped found the Information Access Alliance to address bundling, big deals, and mergers in the publishing industry;
- signed on to the Book Industry Statement supporting the Freedom to Read Protection Act, whose aim was to protect patrons' right to privacy, which was under threat from proposed anti-terrorism legislation; and
- endorsed ALA's “Principles for the Networked World,” which emphasized intellectual freedom, privacy, and equity.

And, in 2004, the MLA Board of Directors:

- wrote a “unified MLA letter” supporting the proposed NIH public access policy, and
- approved the MLANET Editorial Board's use of Creative Commons licensing for the Professional Tool Box [2020 MLA policies, provided by MLA staff].

That was a lot to accomplish in just two years. Interestingly, it was not until 2004 that the first article to use the term “open access” appeared in *JMLA*. Plutchak wrote an editorial, “Embracing Open Access,” which is relevant to this day (and worth reading again). He persuasively argued that “The reason to embrace the open access movement is that it promises to be a very good thing for society, not that it will be a good thing for libraries” [41].

I do not need to tell this audience about the massive impact of the NIH public access policy on open access in biomedicine. In 2008, the NIH public access policy became a mandate rather than a voluntary program. And in 2014, NIH began enforcement by delaying continuing grants for noncompliance [42]. With the first government funder's open access mandate in the United States, helping researchers comply with the policy has in many ways shaped the approach of libraries to supporting open access.

As you can imagine, medical librarians were really questioning relationships with publishers. Going back to 2004, I will never forget the day that Wayne J. Peay, FMLA, made a motion from the floor at the Business Meeting in Washington, DC, “to cease acceptance of funding from publishers that do not support open access publishing.” This prompted considerable discussion, with the more idealistic of us in support. In the end, it was referred to the MLA Board, which at its meeting following the annual meeting, stated “that due to tremendous financial and legal implications, the lack of clarity of what constitutes open access, and recent developments in Congress and NIH which may change the relationship of all publishers to open access, the MLA Board of Directors does not approve or support the motion to cease acceptance of funding from publishers that do not support open access publishing.” On the positive side, the board also stated “that MLA should continue to carefully monitor the situation over the coming year working closely with the Governmental Relations Committee and the Scholarly Publishing Task Force, and encourage members to support open access initiatives and encourage publishers to move to more immediate and reasonable access to their publications” [2020 MLA policies, provided by MLA staff].

In 2005, Plutchak wrote about the impact of open access on the association's journal. He reported that usage statistics showed that 20,000 unique users were accessing the journal every month, and that NLM actually estimated the number of unique users was closer to 30,000, due to shared Internet protocol (IP) addresses. This compared to the membership of MLA, which was 4,500 at the time, and the print run of the journal, which was only 5,000, demonstrating a significant increase in the impact of medical librarian research. However, he identified a future challenge for MLA, which received $200,000 per year in subscriptions and advertising from the journal, a significant source of association revenue. He then asked:

But has the attempt been worth it so far? I look again at the PMC statistics. Twenty to thirty thousand unique users? Has it been worth it? Oh, yes! [43]

From 2007 to 2011, the Ad Hoc Committee for Advocating Scholarly Communications took on the task “to educate and support MLA members, and provide leadership within the association on the broad trends and issues within scholarly communication, including, but not limited to, public access, open access, and the publishing industry.” Finally, at long last, in 2012 MLA established a full-blown Scholarly Communications Committee, which is now planning to become an MLA caucus.

In 2008, the MLA Board of Directors endorsed Health Care Information For All and its vision of “a world where every person will have access to the healthcare information they need to protect their own health and the health of those for whom they are responsible.” And in 2017, MLA joined Re:Create: Supporting a Pro-Innovation, Pro-Creator, Pro-Consumer Copyright Framework [2020 MLA policies, provided by MLA staff].

In that same year, AAHSL worked with the Association of American Medical Colleges and publisher groups to create the Chicago Collaborative, a working group to promote open communication and education among the primary stakeholders in the scholarly scientific communication arena [44].

Most recently, this past fall, the *JMLA* editorial team decided “it is time to ‘practice what we preach,' the *JMLA* is taking a step forward and implementing a firm data sharing policy to increase the rigor and reproducibility of published research, enable data reuse, and promote open science” [45]. To the best of my knowledge, this is the first mandate for data sharing by a peer-reviewed library journal, though many encourage data sharing. However, I was a bit dismayed that the editorial team began the announcement with the statement “librarians are *generally* advocates of open access and data sharing” [emphasis added]. One of my goals is to remove the word “generally” from that statement.

## THE UNIVERSITY OF CALIFORNIA, SAN FRANCISCO (UCSF) EXPERIENCE

I now discuss some of the things that induced me to consider moving to the University of California, San Francisco (UCSF). Before she retired, Karen Butter, FMLA, encouraged me to apply and described the leadership role that she and UCSF had taken over the years in promoting open access.

Way back in the late 1990s, or maybe it was the early aughts, Gail Persily tried to convince me to apply for a job with the UCSF Library. They were launching a new project to create a digital library of tobacco documents. While I chose to stay in Chicago at the time and did not submit an application, I followed the project over the years.

The story begins in 1994, when a set of confidential documents from the Brown & Williamson Tobacco Corporation was leaked to Dr. Stanton Glantz, a cardiologist at UCSF. He deposited the materials in the UCSF Library Archives & Special Collections to protect them and make them available to other researchers. Naturally, the tobacco companies sued. I am happy to report that the University of California (UC) backed up the researchers and the library and won a judgment in favor of the public's right to know. The documents served as a resource for litigators in the multistate lawsuit and master settlement agreement (MSA), reached in November 1998 between the state attorneys general of forty-six states, five US territories, the District of Columbia, and the four largest cigarette manufacturers in America.

Following this major victory for public health, my predecessor Butter suggested putting the original documents, as well as those released as a result of the MSA, on the Internet to make them available to researchers around the world. The result was the Legacy Tobacco Documents Library, now part of the open access Industry Documents Library, which is still growing and includes documents from the chemical, drug, food, and fossil fuel industries that influence public health [46]. The more than 93 million pages in over 15 million documents have been cited in more than 1,000 publications [47]. It is no understatement to say that the efforts of the UCSF Center for Tobacco Control Research and Education have saved millions of lives, and medical librarians and archivists were there from the beginning.

Open access has been adopted by all areas of medical librarianship. When I arrived at UCSF, I learned about the Medical Heritage Library (MHL), founded in 2010 by a consortium of medical libraries to provide open access to history of medicine and health resources. In collaboration with the Internet Archive, the members have digitized historical American journals, primary resources in topic areas such as vaccines and racism in health and medicine, and hundreds of thousands of rare medical books, pamphlets, journals, and films. UCSF was honored to be invited to join as a member in 2018, following our participation in a project to digitize every state medical journal in the United States, Washington, DC, and Puerto Rico (except Massachusetts, which has the *New England Journal of Medicine,* and New Hampshire, which never had a medical journal). Jisc and the Wellcome Library have also sponsored the UK Medical Heritage Library, focused on nineteenth and early twentieth century history of medicine and related disciplines [48].

Of course, I was already aware that the UCSF Academic Senate implemented a policy to make research papers freely accessible to the public in 2012. The press release stated: “The unanimous vote of the faculty senate makes UCSF the largest scientific institution in the nation to adopt an open-access policy and among the first public universities to do so” [49]. This was the result of years of work by librarians to educate the academic community at UCSF about open access—work that many of our fellow librarians have undertaken at their own institutions.

A year later, spurred by UCSF, the systemwide UC Faculty Senate passed a nearly identical policy [50]. In 2015, UC President Janet Napolitano issued a presidential open access policy that expanded open access requirements to all authors who were not covered by the faculty senate policies [51]. However, these groundbreaking policies have not yet resulted in the change we would like to see. Five years later, we are just now implementing the presidential open access policy in eScholarship, the UC institutional repository. As one of our faculty members said a few years back (paraphrased): “We've been at this for 15 years, and only 15% of peer reviewed literature is open access from the date of publication. At this rate, it will take 85 more years to realize the vision. We can't wait that long!”

At UC, we believe we are at a tipping point, and now is the time. The UC Project Transform Working Group, a collaboration of librarians and faculty, is working to flip existing scholarly publishers to open access. The recently announced deal with Springer-Nature is the first of what we hope will be many such “transformative agreements” [52]. We are committed to working with our community and both commercial and open access publishers to make it a reality. If you want to learn more, an immersion session during the MLA '20 vConference provides more information.

## OPEN SCIENCE

And here we are today, at the first MLA vConference, in the middle of two pandemics: COVID-19, caused by a coronavirus, and anti-Black violence, caused by racism in our society. Both are urgent public health crises that deserve our attention as medical librarians.

We have responded to crises in the past, most notably in World Wars I and II. In 1917, Grace Myers created a “war bibliography” that was published in the *BMLA,* distributed to “every camp and cantonment in the United States,” and used as a source for the *War Supplement of the Index Medicus* [52]. In the early 1940s, medical librarians once again responded, providing services to all medical officers and hospitals, and sending books and other materials to medical camps. Medical libraries and librarians supported the work of libraries in Europe and shared duplicate subscriptions [54]. And I remind you of Jerry's work during the HIV/AIDS pandemic, highlighted in last year's Janet Doe Lecture. Now, in the midst of the COVID-19 pandemic, we see publishers responding to a call from the Wellcome Trust to lower the paywall and provide free (but not open) access to publications related to COVID-19. Medical librarians are spreading the word about NLM initiatives like the COVID-19 Open Research Dataset (CORD-19) and the NIH Preprint Pilot in PubMed Central. All aspects of research related to COVID-19 are moving at an incredible pace. As Dr. Esther Choo said in the John P. McGovern Award Lecture during the MLA '20 vConference, we are experiencing “science on steroids.”

Medical librarians must apply the lessons learned from these experiences to all aspects of biomedical research to achieve our vision. We recognize that this will be a complicated journey. For example, if we are not careful, the focus on article processing charges (APCs) as a funding mechanism for scholarly publishing might perpetuate the inequities of the current system, as researchers who lack funding will encounter new barriers to publishing. And, as Choo so compellingly demonstrated, open science does not, by itself, reduce health care disparities.

So, what is open science? It is the natural next step beyond open access. UNESCO defines open science as “the movement to make scientific research and data accessible to all” [55]. The Organisation for Economic Co-operation and Development (OECD) says: “Broadening access to scientific publications and data is at the heart of open science, so that research outputs are in the hands of as many as possible, and potential benefits are spread as widely as possible” [56]. The FOSTER Open Science Taxonomy identifies six major elements of open science: open access, open data, open reproducible research, open science evaluation, open science policies, and open science tools [8].

Open Science Is the Future of Research!

Medical librarians have been leaders in improving access since 1898. Take the next step on that journey and join the conversation at the open science session, “Roles to Play: Open Science & Health Sciences Librarians,” during the MLA '20 vConference.

Thank you.
